# Metabolomic Profiling Unveils the Impact of Non-Doped and Heteroatom-Doped Carbon Nanodots on Zebrafish (*Danio rerio*) Embryos

**DOI:** 10.3390/nano11020483

**Published:** 2021-02-14

**Authors:** Theodoros G. Chatzimitakos, Claire Pliatsika, Ieremias Chousidis, Ioannis D. Leonardos, Constantine D. Stalikas

**Affiliations:** 1 Laboratory of Analytical Chemistry, Department of Chemistry, University of Ioannina, 45110 Ioannina, Greece; chatzimitakos@outlook.com (T.G.C.); claire.dchemrid@gmail.com (C.P.); 2 Laboratory of Zoology, Department of Biological Applications and Technologies, University of Ioannina, 45110 Ioannina, Greece; jchousidis@gmail.com (I.C.); ileonard@uoi.gr (I.D.L.)

**Keywords:** zebrafish, metabolomics, non-doped carbon nanodots, N-doped carbon nanodots, N,S-codoped carbon nanodots

## Abstract

Recently, concern has been raised over the transport, transformation, and fate of carbon nanodots (CNDs) after their release into the environment. Their toxicity towards organisms and humans has recently been addressed as an important issue. In this study, a metabolomic approach was employed to obtain an insight into the effect of CNDs (either pristine or doped with nitrogen and nitrogen/sulfur) on zebrafish. Embryos were exposed to concentrations corresponding to lethal concentration (LC) LC_50_ (550, 400, and 150 μg mL^−1^), LC_50/2_ (275, 200, and 75 μg mL^−1^), and LC_50/4_ (138, 100, and 38 μg mL^−1^) of the three CNDs (non-doped, N-doped, and N,S-codoped, respectively) to scrutinize the interactions of the CNDs with the larvae. Numerous differences in the metabolic pathways were recorded in all cases. Seven metabolic pathways were detected in the control larvae. When the larvae were exposed to concentrations equal to LC_50_, LC_50/2_, and LC_50/4_ of non-doped CNDs, 12, 12, and 3 metabolic pathways were detected, respectively. In the case of N-doped CNDs, 4, 7, and 4 pathways were detected, while in the case of N,S-codoped CNDs, 8, 5, and 5 pathways were detected when exposed to concentrations of LC_50_, LC_50/2_, and LC_50/4_, respectively. In all cases, certain metabolic pathways were altered while others were either down-regulated or up-regulated. Some of these changes include the activation of alanine, aspartate, and glutamate metabolism, aminoacyl-tRNA biosynthesis, butanoate metabolism, D-glutamine, and D-glutamate metabolism, glutathione metabolism, selenoamino acid metabolism, valine, leucine, and isoleucine degradation pathways. Moreover, the deactivation of starch and sucrose metabolism, the glycine, serine, and threonine metabolism, among others, were recorded. Our findings underline the importance to further study the impact of CNDs on marine organisms. As zebrafish has been shown to share many similarities with humans in bioprocesses and genome, it can be assumed that CNDs may also pose a threat to human health.

## 1. Introduction

The research interest involving nanomaterials continues to grow unabated as more and more researchers have been involved in this research field, aiming to exploit the expanding range of properties of nanomaterials [1]. The advancement in the development of innovative nanomaterials has led to the synthesis of carbon nanodots (CNDs). The research interest around CNDs not only gave birth to a huge number of studies but also established a new, flourishing field of research. Since the discovery of CNDs over a decade ago, research on their synthesis and application has grown exponentially, and enormous interest has been aroused in introducing them into health-, environmental-, and technological-related applications [2,3,4,5]. It was not until recently that CNDs were used as antibacterial agents, fluorescent agents in cell imaging applications, drug carriers, and probes for the in vitro and in vivo detection of specific molecules, and they have even been proposed for human cancer theragnostic and phototherapy applications [6,7,8,9,10]. Owing to their enthralling features, CNDs are considered to be the next-generation platform for biomedical applications [11]. Another great feature of CNDs is their potential for heteroatom doping. The CNDs can easily be doped either with single or multiple elements, simultaneously. The CNDs doped with nitrogen or codoped with nitrogen and sulfur have been widely used so far. Moreover, doping with transition metals has gained interest during the last years, widening, even more, their applicability [12,13].

However, what would seem a highly promising and propitious material can backfire if used hastily without first examining its potential side effects [14,15]. Obtaining fresh scientific evidence for the safe use of CNDs for human health-related and other application-relevant extensive studies are called for. Considering that (i) the published articles which refer to CNDs as materials of low toxicity and/or biocompatible, by conducting experiments almost solely in eukaryotic cells, (ii) there is a need to shed light on all the interactions that occur in more complex organisms, and not only at a cellular level, and (iii) the widespread use of CNDs will inevitably result in a discharge into the environment, which can raise environmental safety issues for micro and macroflora, fauna, and ultimately biota, it is imperative that relevant in-depth research on the effects of CNDs on advanced organisms should be done. 

Up to now, such studies are scanty and sparse. The first study regarding the toxicity of graphene quantum dots was published in 2015, in which, decreased heart rate, pericardial edema, bent tail, and alterations in the activity of embryonic zebrafish were reported [16]. Another study was published where relevant findings were recorded on rare minnow embryos exposed to CNDs [17]. Dias et al. pointed out that CNDs synthesized from different fruits (used as precursor material) exhibited different toxicity and caused various phenotype alterations on zebrafish embryos and hinting that the elemental composition of CNDs can alter their effect [18]. To examine whether this is the case with several CNDs, our team examined the effect of non-doped, nitrogen-doped (N-doped), and nitrogen/sulfur codoped (N,S-codoped) CNDs on zebrafish [19]. From the results, it appeared that all CNDs exhibit moderate toxicity (in many cases, phenotype alterations were also recorded), which was not only concentration-dependent but also dopant-dependent. The more the heteroatoms used to dope CNDs, the higher the mortality and the more the severe malformations caused to zebrafish by the respective CNDs species. Even though the above studies examined the effect of CNDs in more complex organisms, interactions of CNDs with the tested organisms at the metabolite level were not elucidated, resulting in a lack of knowledge on the topic.

This study aims to obtain a better insight into the metabolic alterations that occur in zebrafish upon their exposure to CNDs and may be responsible for the phenotypic alterations. To this end, a metabolomic approach was adopted, since it is the most relevant field that correlates the pools of biological molecules with the function/dynamics of an organism [20]. Zebrafish were exposed to different concentrations of non-doped, N-doped, and N,S-codoped CNDs, which were synthesized using citric acid as a carbon source and other molecules for doping elements, based on previous findings [19]. To scrutinize the alterations of metabolomes from exposed and non-exposed zebrafish, an untargeted approach was followed. Plenty of differences were recorded in the metabolomes of zebrafish which can also explain the observed phenotype alterations. 

## 2. Materials and Methods 

### 2.1. Chemicals

Citric acid, urea, thiourea, benzoylated dialysis tubing membrane (MWCO: 2000 KDa), and all solvents were obtained from Sigma-Aldrich (Hellas, Greece). Deuterated water (D_2_O) and 3-(trimethylsilyl)-1-propionic-2, 2, 3, 3-d4 acid sodium salt (TSP) were obtained from Deutero (Deutero GmbH, Kastellaun, Germany).

### 2.2. Instrumental Conditions

All information regarding the instrumentation used in this study is given in supplementary information.

### 2.3. CND Synthesis and Characterization

The CNDs used in this study were synthesized according to our previous studies [19,21]. In brief, for the synthesis of the non-doped CNDs, 0.20 g of citric acid was heated in a stainless steel Teflon-lined autoclave at a rate of 10 °C min^−1^ at 200 °C, for 3 h. For the synthesis of the N-doped CNDs, 0.13 g of citric acid and 0.11 g of urea were dissolved in 3 mL of double distilled water to form a transparent solution, which was heated in an autoclave at 160 °C, for 8 h. For the synthesis of the N,S-codoped CNDs, 0.10 g of citric acid and 0.11 g of thiourea were homogenized in a mortar and the mixture was heated in an autoclave at 200 °C, for 2 h. To the resulting sticky liquids, double-distilled water was added, and the mixtures were centrifuged at 4000 g. Subsequently, the pH values of the supernatants were adjusted to 7.0 and were dialyzed against water for 24 h (changing the water every 3 h) using a benzoylated dialysis tubing membrane (MWCO: 2000 KDa). 

The synthesized CNDs were characterized by obtaining their FTIR spectra and HRTEM images, and measuring their zeta potential. Details can be found in the supporting information.

### 2.4. Exposure of Zebrafish to CNDs

All experiments conformed to the European Directive 2010/63. In this study, adult zebrafish of the wild type (AB) were maintained at 28 °C with a photoperiod of 14 h light/10 h dark and fed twice a day. Sexually mature zebrafish were used for spawning. Eggs were collected and placed in E3 embryo medium (5 mmol L^−1^ NaCl, 0.33 mmol L^−1^ CaCl_2_, 0.33 mmol L^−1^ MgSO_4_ and 0.17 mmol L^−1^ KCl). After 24 h post fertilization (hpf) eggs were dechorionated (dechorionation started before to ensure that fertilized dechorionated embryos were ready for exposure, at exactly 24 hpf). Embryos were dechorionated using two forceps with sharp tips [22]. The duration of each experiment was set at 96 h [23], beginning at 24 hpf (to ensure the viability of zebrafish embryos), at 26-somite point, according to Kimmel [24]. Two embryos were placed in each well of a 24-well plate containing 1.5 mL E3 solution. Zebrafish were exposed to three concentrations (i.e., LC_50_, LC_50_/_2_ and LC_50_/_4_) of each CNDs species (i.e., 550, 275, and 138 μg mL^−1^ non-doped CNDs, 400, 200, and 100 μg mL^−1^ N-doped CNDs and 150, 75, and 38 μg mL^−1^ N,S-codoped CNDs). After 96 h, embryos were collected and washed several times with PBS, and then euthanized by snap freezing with liquid nitrogen. This also assists to quench the metabolism of the larvae. Then, the samples were freeze-dried to remove water (and further assist in the extraction of metabolites), and used for further experiments [19]. The experiments were repeated three times, and each time 240 embryos were used for each tested concentration. 

### 2.5. Metabolome Extraction and Analysis

The metabolomes of zebrafish larvae were extracted according to a previously described procedure [25]. In brief, chloroform (1 mL) and methanol (2 mL) were added into the lyophilized zebrafish larvae, the mixture was freeze-thawed three times using liquid nitrogen and was ultrasonicated for 3 min. No other homogenization technique was employed, as it was not found important for obtaining better metabolome extracts [26]. Then, 1 mL of chloroform and 1 mL of water were added, and the mixture was vortexed for 30 sec. The phases were separated by centrifugation at 2000 g for 5 min. The upper phase was collected and divided into two equal portions, which were evaporated to dryness, under nitrogen. To obtain the ^1^H-NMR spectra, one of the two residues was resuspended in 600 μL of D_2_O (containing TSP) and the new solution was transferred to a 5-mm NMR glass tube. The second residue was resuspended in 100 μL of acetonitrile for further analysis using LC-HRMS (Thermo Scientific, Bremen, Germany).

### 2.6. Metabolome Data Processing

The processing of the obtained spectra was carried out according to our previous studies [25,27,28]. In brief, the Human Metabolome Database (www.hmdb.ca) and the Madison Metabolomics Consortium Database (www.mmcd.nmrfam.wisc.edu) were used for the analysis of the NMR spectra. The presence of the tentative metabolites that resulted from the above analysis was further confirmed by scanning the MS spectra for the accurate masses (to 4 decimals) of the metabolites and comparing fragmentation patterns with data from online databases. Metabolic pathways were obtained using the pathway analysis tool of the MetaboAnalyst suite (www.metaboanalyst.ca) using the *Danio Rerio* metabolic pathway library. 

## 3. Results

All information regarding the characterization of the CNDs is presented in Supporting Information (Section 2 and Figures S1–S7). The characterization showed that the three CNDs have an average size of ~5 nm (Figure S1). Doping with heteroatoms is confirmed with the FTIR spectra, in which characteristic peaks for nitrogen- and sulfur-containing groups can be seen (Figures S2–S4). Finally, based on the zeta potential measurements, it can be concluded that at pH = 7, the N-doped and N,S-codoped CNDs exhibit from good to high colloidal stability and are negatively charged, to a different degree (Figures S5–S7). Based on the above, the three CNDs exhibit structural differences, which are expected to alter their properties (including their toxicity). 

**Figure 1:**
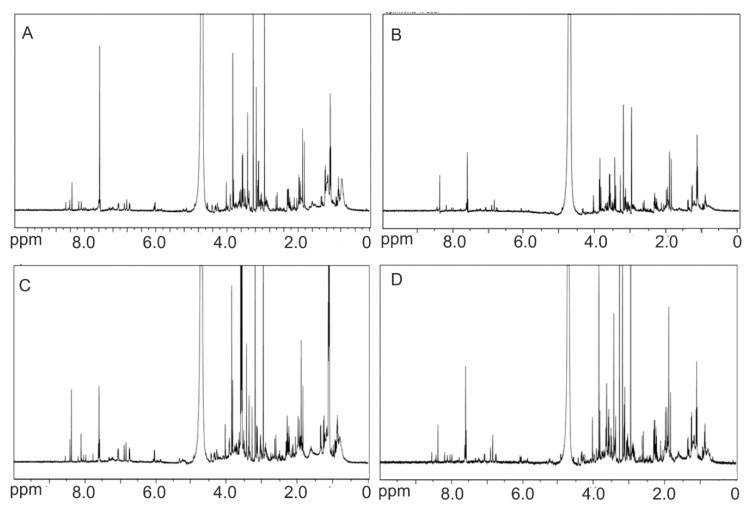
^1^H-NMR spectra of (**A**) control zebrafish and zebrafish treated with LC_50_ concentration of (**B**) non-doped, (**C**) N-doped, and (**D**) N,S-codoped CNDs.

Based on previous results, the three kinds of CNDs exhibit different mortality to zebrafish larvae [19]. The LC_50_ values of the three nanomaterials are 550 (non-doped), 400 (N-doped), and 150 (N,S-codoped) μg mL^−1^. Our experiments were carried out at three concentrations, i.e., at LC_50_, LC_50_/_2,_ and LC_50_/_4_, to acquire data on metabolic alterations under conditions consistent with the concentration-dependence of the three CNDs species. Representative ^1^H-NMR spectra of the obtained metabolomes can be seen in Figure 1. As can be seen, most differences are recorded in the region of 2.5–4.5 ppm where mostly hydrogen from alcohols and carbohydrates give a resonance signal. Moreover, differences can be seen in the region of 6.0–9.0 where the most signals of the most deshielded protons (such as aromatic and carboxylic) are. Next, the metabolites for each sample were identified. The information about all the detected metabolites can be found in the Supplementary Information (Tables S1 and S2).

### 3.1. Metabolomic Study of Non-doped CNDs Effect

**Figure 2:**
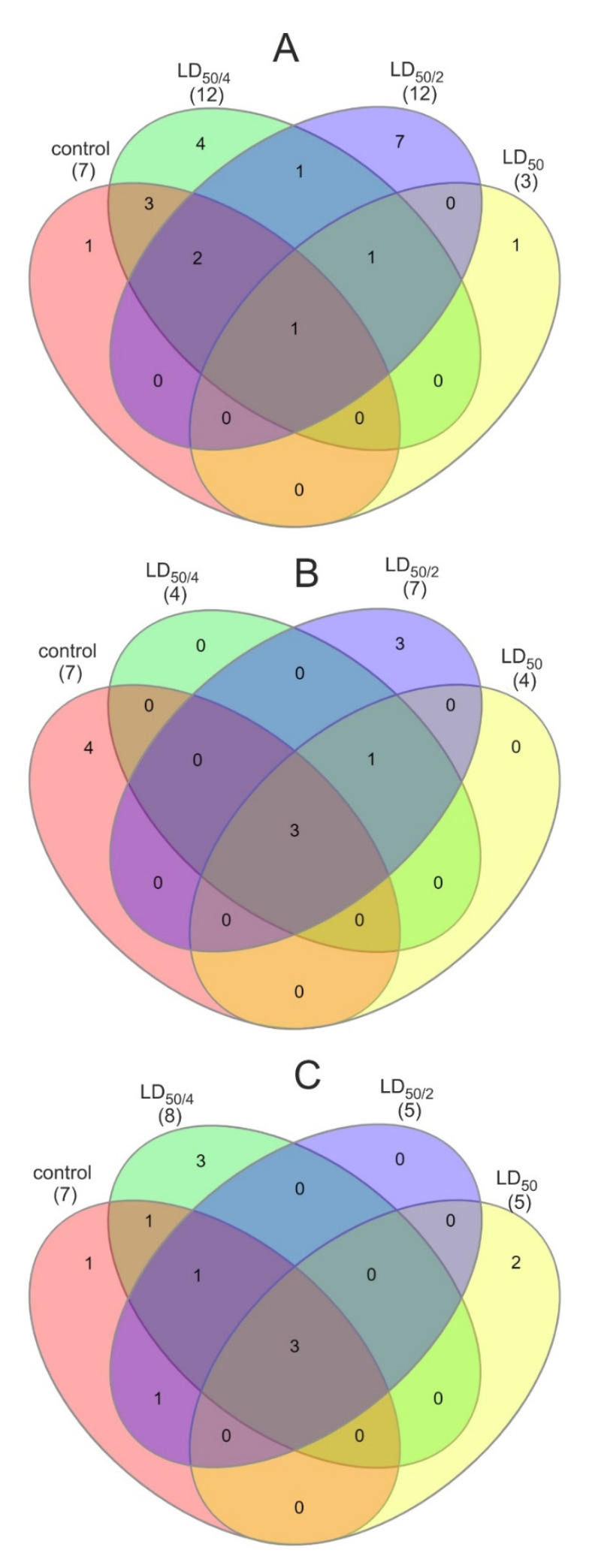
Venn diagrams of control and all tested concentrations of (**A**) non-doped, (**B**) N-doped, and (**C**) N,S-codoped CNDs; red denotes the control sample, green the sample treated with LC_50/4_, blue the sample treated with LC_50/2_, and yellow the sample treated with LC_50_.

When zebrafish larvae were exposed to non-doped CNDs, several alterations in the metabolome were observed (Table S3). Seven metabolites (mostly carbohydrates) were absent in the treated samples, while some others, such as biotin and lactose, were absent in the metabolomes of larvae treated with the two highest concentrations of CNDs. At the lowest of the tested concentration (LC_50/4_), eight new metabolites emerged in the sample, i.e., citrulline, gamma-aminobutyric acid, glutathione, fucose, histidine, tyrosine, melibiose, and selenomethionine. Likewise, in the second tested concentration (LC_50/2_), six new metabolites appeared, i.e., alpha-ketoisovaleric acid, inosine, arabitol, cystine, pipecolic acid, and trehalose. On the contrary, the metabolome of larvae treated with the highest concentration (LC_50_) did not contain any new metabolites. The metabolic pathways that are associated with the above metabolites can be seen in Table 1. It is obvious that the pathways of starch and sucrose metabolism were not affected, in all cases, whereas the riboflavin metabolism was down-regulated. At the concentration of LC_50_/_2_, the pathways of amino sugar and nucleotide sugar metabolism, fructose and mannose metabolism, and biotin metabolism were also down-regulated, while at the LC_50_ concentration, the above pathways along with the galactose and nitrogen metabolism pathways were down-regulated. The number of common pathways among samples is visually described using a Venn diagram (Figure 2).

### 3.2. Metabolomic Study of N-doped CNDs Effect

Larvae exposed to N-doped CNDs exhibited a metabolic profile different from the above. At all tested concentrations, five metabolites remained unchanged (mostly carbohydrates) while nine others (fructose 6-phosphate, glucosamine 6-phosphate, D-mannose, uridine diphosphate glucuronic acid, uridine diphosphate-N-acetylglucosamine, D-fructose, biotin, L-cystathionine, and sucrose) were not detected in any of the treated samples (Table S4). Interestingly, only one new metabolite was detected in all treated samples (S-adenosylhomocysteine), and three more metabolites (isoleucine, xylose, and citrulline) were detected only in the metabolome of larvae treated with LC_50/2_ N-doped CNDs. With respect to metabolic pathways (Table 2), three of them remained unaltered in all cases (starch and sucrose metabolism, galactose metabolism, amino sugar, and nucleotide sugar metabolism) while four others were down-regulated (nitrogen metabolism, fructose, and mannose metabolism, biotin metabolism, and riboflavin metabolism). Moreover, in all treated samples, the cysteine and methionine metabolism pathways were up-regulated. 

### 3.3. Metabolomic Study of N,S-codoped CND Effect

When zebrafish were exposed to N,S-codoped CNDs only maltose and glucosamine-6-phosphate remained unaltered. Uridine diphosphate glucuronic acid, biotin, fructose, and uridine diphosphate-N-acetylglucosamine were not detected in any of the samples. The two lowest concentrations exhibited similar profile whereas only three metabolites (lactose, maltose, and glucosamine 6-phosphate) were detected in the metabolomes of larvae exposed to the highest concentration, also existing in the control sample (Table 3). The S-adenosylhomocysteine, opposite to the N-doped CNDs, was detected only in the metabolome of larvae exposed to the concentration of LC_50_, while at the two other concentrations, three (at LC_50/2_) and four (at LC_50/4_) new metabolites were detected, which were absent in the control sample. The starch and sucrose metabolism, galactose metabolism, and the amino sugar and nucleotide sugar metabolism were the metabolic pathways not affected in all cases (Table 3). The riboflavin metabolism pathway was down-regulated in all cases. The pentose and glucuronate interconversions, valine, leucine and isoleucine biosynthesis, and the aminoacyl-tRNA biosynthesis were up-regulated in the lowest concentration employed, while the cysteine and methionine metabolism and the alanine, aspartate, and glutamate metabolism were up-regulated at the highest examined concentration. 

## 4. Discussion

In all cases examined, numerous metabolic alterations were observed between the different materials and among the different concentrations of the same material. This is consistent with our previous study, where the observed phenotypic alterations of zebrafish (e.g., pericardial edema, yolk sac edema, and tail and spine curvature) were dependent on the concentration and the type of the material used [19]. The differences observed in the metabolic changes are attributed to the heteroatom doping that bestows CNDs with different physicochemical properties. It is known that the size of nanomaterials is a crucial parameter that affects their toxicity [29]. As can be seen in Figure S1, the size of the three CNDs and their size distribution are almost the same. Therefore, these factor cannot be determining to support the differences in the metabolomes. Moreover, all three CNDs have the same circular shape, so alterations cannot be attributed to this parameter either. The surface charge of the nanomaterials is an important parameter. From Figures S5–S7, it can be seen that the studied CNDs have a negative or negative/neutral surface charge. It is also known that nanoparticles with neutral surface charge are more biocompatible compared with the negatively charged ones [29]. The non-doped CNDs exhibit an almost neutral surface charge and as such, they are expected to exhibit the lowest mortality, which is in accordance with the experimental LC_50_ values. Since the non-doped CNDs are more biocompatible, organisms exposed to them maintain the metabolic pathways that can be used to counterbalance their effect. This was plausible in our study because the most differences in the number of active metabolic pathways were observed in the case of non-doped CNDs compared with the two other nanomaterials. With respect to the N-doped and N,S-codoped CNDs, the latter has a higher (negative) surface charge compared with the former, but the number of the active metabolic pathways of zebrafish was comparable for both. Hence, the composition of the nanomaterials contributes to such differences [30]. The different functional groups on their surface make feasible the interactions with different molecules. This is strengthened by the different hydrogen donor behavior of the CNDs, which also accounts for their different antioxidant properties [21,30,31]. In any case, examining the metabolic changes can yield much more information about the existing phenotypic alterations and future potential health-related issues.

### 4.1. Glutathione Metabolism

Glutathione exhibits significant antioxidant activity in organisms. Due to its significant role in the deactivation of reactive oxygen species, it is usually synthesized in ubiquitous amounts to cope with oxidative stress [32]. In our case, glutathione metabolism was stimulated only in the zebrafish exposed to non-doped CNDs. Therefore, the exposure of larvae to non-doped CNDs induces oxidative stress. Although the two other kinds of CNDs are more harmful to the larvae (according to the LC_50_ values), the pathway of glutathione metabolism was not found to be up-regulated in any of the other samples. This can be attributed to the inherent antioxidant potency of these materials. In a previous study, we found that the N-doped CNDs exhibit the highest antioxidant activity (even higher than BHT or gallic acid in some cases), followed by the N,S-codoped CNDs, in contrast to the non-doped CNDs which exhibit negligible antioxidant activity [33]. Therefore, it is reasonable that although all three kinds of CNDs induce oxidative stress to zebrafish larvae, the stress from N-doped and N,S-codoped CNDs is counterbalanced by their considerable antioxidant properties. The phenotypic alterations in the cases of N-doped and N,S-codoped CNDs cannot be attributed to the oxidative stress, while in the case of the non-doped CNDs, this is one of the primary reasons for the observed phenotypes.

### 4.2. Glycerolipid and Glycerophospholipid Metabolism

Lipids are the building blocks of cell membranes and neural tissues, while they can serve as energy sources [34]. Glycerolipids are a group of lipids that have vital structural and functional roles in cell membranes. Glycerophospholipids are amphiphilic lipids and are found abundantly in eukaryotic cells [35]. After exposure of zebrafish to non-doped CNDs, the metabolism of glycerolipids and glycerophospholipids was up-regulated, compared with the control sample; however, this was not the case with the two other nanomaterials. The activation of this pathway is associated with the higher oxidative stress that larvae are subjected to, as stated above. Lipids that are present in cell membranes are a common target of free radicals, thus leading to lipid peroxidation. This causes damages to the cell membranes and hence, dysfunction of the cells themselves [34]. Consequently, lipids in the cell membranes of larvae previously exposed to non-doped CNDs may have been oxidized. The metabolism of glycerolipids and glycerophospholipids was, then, activated to repair the damage to the cell membranes. 

### 4.3. Riboflavin Metabolism

Riboflavin is the precursor of flavin mononucleotide (FMN) and flavin adenine dinucleotide (FAD) [36,37]. Changes in the riboflavin metabolism were observed in all cases, compared with control samples. This results in low concentrations of FMN and FAD in the interior of cells, giving rise to reduced activity, primarily, of acyl-CoA-dehydrogenases, thus leading to impaired fatty acid oxidation. Moreover, increased protein carbonylation and breakage of the DNA strand are attributed to riboflavin deficiency [36]. 

### 4.4. Biotin Metabolism

Biotin acts as a coenzyme of carboxylases that take part in the normal metabolism of proteins, lipids, and carbohydrates. It is necessary for the normal progression of gluconeogenesis, fatty acid synthesis, and amino acid catabolism pathways, and is vital for the expression of some genes [38]. More importantly, biotin is associated with the induction of the receptors for yolk binding proteins. Biotin metabolism was down-regulated in zebrafish under nearly all tested conditions. Therefore, a biotin deficiency is expected, which can result in poor performance of the yolk binding protein receptors. As a consequence, a malabsorption of nutrients from the yolk sac is anticipated. Since the yolk sac is vital (at this developmental stage) for the survival and the development of the embryos, reduced growth rates and development are expected for larvae exposed to CNDs. This was verified by our morphological observation study [19] and is in agreement with previous studies [39,40]. Moreover, other health conditions have been associated with biotin deficiency, such as increased mortality, skin, and gill abnormalities, and convulsions [39,40].

### 4.5. Nitrogen Metabolism

In a previous study, it was suggested that dysfunction of the nitrogen metabolism pathway can be attributed to problems in the normal function of the gills [41]. As can be inferred from our results, the nitrogen metabolism pathway was down-regulated at all the concentrations of N-doped CNDs examined, as well as at the highest tested concentrations of the two other species. Therefore, it can be concluded that all CNDs can induce damage to zebrafish gills at high concentrations; however, such damage is caused by the N-doped dots at lower concentrations.

### 4.6. Glucose Alterations—Energy Metabolism

Glucose is one of the most important carbohydrates for living organisms, as it is the primary source of energy for cells. As shown in Tables S3–S5, the metabolite α-D-glucose, present in the control sample, disappears from the metabolomes of zebrafish larvae after they have been exposed to all tested concentrations of non-doped CNDs, as well as to the LC_50_ concentration of N,S-codoped CNDs. This indicates the creation of disturbances to the normal course of glycolysis/gluconeogenesis, which can lead to reduced energy levels in the larvae, making it difficult for them to survive [25,41]. Glycolysis/gluconeogenesis occurs mainly in the liver. The disturbance of glucose levels reflects glycolysis/gluconeogenesis disorders, which underline possible liver damage to the larvae, after exposure to CNDs. Such damages can also be responsible for the observed mortality. Interestingly, after exposure to N,S-codoped CNDs at a concentration equal to LC_50_, the activation of alanine, aspartate, and glutamate metabolism was recorded without detecting glutamate. The latter can serve as an energy source; since glutamate was not detected (despite the activation of the respective pathway), it implies that it served as an energy source [41]. 

### 4.7. Amino Acid Metabolism

During the early development and given that the organogenesis takes place, amino acids are essential. When zebrafish were exposed to CNDs, essential amino acid-related pathways were up-regulated. The emergence of these metabolic pathways, therefore, hints towards muscle damage of the larvae after exposure to CNDs, so that essential amino acids can result from the breakdown of the muscle proteins [25]. Muscle breakdown may be associated with changes in biotin levels, as stated above, since the necessary amino acids cannot be absorbed from the yolk sac. Moreover, as the amino acid-related pathways were up-regulated, mostly in the larvae exposed to non-doped CNDs, muscle breakdown may be attributed to the oxidative damage to proteins. Therefore, the growth rate was reduced in a concentration- as well dopant-dependent manner.

Multitudinous studies have demonstrated, so far, the biocompatibility of CNDs and their low cytotoxicity using eukaryotic cell models. However, only a few reports have examined the effect of CNDs on more advanced organisms, and even fewer have explored their effect at a metabolic level. According to all the results presented herein, the CNDs can significantly alter the metabolism of zebrafish. Multiple alterations can take place, which result in a distorted phenotype and can lead to acute toxicity. Furthermore, these alterations hint towards the development of other health issues, at a later developmental stage of the larvae. As can be seen from the results, most metabolic alterations were observed in the case of non-doped CNDs, followed by N,S-codoped CNDs and the N-doped CNDs. Although the non-doped CNDs cause the lowest mortality (since the LC values are higher than those of the other CNDs) they cause the most metabolic alterations and can potentially cause more dysfunctions in the long-term. Based on the above, the CNDs pose a threat to zebrafish and even more can threaten other marine organisms, and humans (zebrafish are considered as a representative vertebrate model that well-resembles human). Moreover, it is made apparent that the metabolomic approach employed herein can be used as a benchmark for the assessment of the metabolic alterations that other nanomaterials induce to organisms, as well as to study the effect of other factors, such as pure compounds [25] or even viruses on the gut [42].

## 5. Conclusions

This study examined the effect of CNDs on the metabolism of zebrafish. The three CNDs, i.e., non-doped CNDs, nitrogen-doped, and nitrogen/sulfur codoped analogs were studied at three concentrations to obtain insights into the metabolic alterations. It is concluded that the exposure of zebrafish to CNDs brings about multiple metabolomic alterations that are responsible for the phenotypic alterations. They were found to be dependent on the different heteroatom dopants of the CNDs and their concentrations. In most cases, new metabolic pathways were up-regulated compared with the control larvae. The up-regulation of some pathways occurred to assist the survivability of the organisms and counterbalance the negative impact of the CNDs. The exposure of zebrafish to CNDs caused many unwanted side effects that can affect the viability, the reproducibility and the overall health of the organism. Most of these phenotypic alterations can be accounted for by the results of this study, highlighting the importance of metabolic alterations due to xenobiotics. As zebrafish is highly homologous to other mammals and has been shown to share many similarities in bioprocesses and genome with humans, it can be concluded that CNDs may also pose a threat to human health. Therefore, more research is needed before large-scale production of CNDs and mainly, before developing human-targeted health-related applications.

## Figures and Tables

**Table 1:** Metabolic pathways of control larvae and larvae treated with 138, 275, and 555 μg mL^−1^ of non-doped CNDs; + denotes active metabolic pathways in the sample, while - denotes down-regulated pathways.

Metabolic Pathway	Control	138 μg mL^−1^ (LC_50/4_)	275 μg mL^−1^ (LC_50/2_)	550 μg mL^−1^ (LC_50_)
Starch and sucrose metabolism	+	+	+	+
Galactose metabolism	+	+	+	-
Nitrogen metabolism	+	+	+	-
Amino sugar and nucleotide sugar metabolism	+	+	-	-
Fructose and mannose metabolism	+	+	-	-
Biotin metabolism	+	+	-	-
Riboflavin metabolism	+	-	-	-
Aminoacyl-tRNA biosynthesis	-	+	-	-
Arginine and proline metabolism	-	+	-	-
Glutathione metabolism	-	+	+	-
Histidine metabolism	-	+	-	-
Selenoamino acid metabolism	-	+	-	-
Pantothenate and CoA biosynthesis	-	-	+	-
Pentose and glucuronate interconversions	-	-	+	-
Purine metabolism	-	-	+	-
Valine, leucine, and isoleucine biosynthesis	-	-	+	-
Valine, leucine, and isoleucine degradation	-	-	+	-
Glycerolipid metabolism	-	-	+	-
Glycerophospholipid metabolism	-	-	+	-
Glycine, serine, and threonine metabolism	-	-	-	+
Cysteine and methionine metabolism	-	+	+	+

**Table 2:** Metabolic pathways of control larvae and larvae treated with 100, 200, and 400 μg mL^−1^ of N-doped CNDs; + denotes active metabolic pathways in the sample, while - denotes down-regulated pathways.

Metabolic Pathway	Control	100 μg mL^−1^ (LC_50/4_)	200 μg mL^−1^ (LC_50/2_)	400 μg mL^−1^ (LC_50_)
Starch and sucrose metabolism	+	+	+	+
Galactose metabolism	+	+	+	+
Amino sugar and nucleotide sugar metabolism	+	+	+	+
Nitrogen metabolism	+	-	-	-
Fructose and mannose metabolism	+	-	-	-
Biotin metabolism	+	-	-	-
Riboflavin metabolism	+	-	-	-
Arginine and proline metabolism	-	-	+	-
Pentose and glucuronate interconversions	-	-	+	-
Cysteine and methionine metabolism	-	+	+	+
Valine, leucine, and isoleucine biosynthesis	-	-	+	-

**Table 3:** Metabolic pathways of control larvae and larvae treated with 38, 75, and 150 μg mL^−1^ of N,S-codoped CNDs; + denotes active metabolic pathways in the sample, while - denotes down-regulated pathways.

Metabolic Pathway	Control	38 μg mL^−1^ (LC_50_/4)	75 μg mL^−1^ (LC_50_/2)	150 μg mL^−1^ (LC_50_)
Starch and sucrose metabolism	+	+	+	+
Galactose metabolism	+	+	+	+
Amino sugar and nucleotide sugar metabolism	+	+	+	+
Nitrogen metabolism	+	+	+	-
Fructose and mannose metabolism	+	+	-	-
Biotin metabolism	+	-	+	-
Riboflavin metabolism	+	-	-	-
Pentose and glucuronate interconversions	-	+	-	-
Valine, leucine, and isoleucine biosynthesis	-	+	-	-
Aminoacyl-tRNA biosynthesis	-	+	-	-
Cysteine and methionine metabolism	-	-	-	+
Alanine, aspartate and glutamate metabolism	-	-	-	+

## Supplementary Materials

The following are available online at https://www.mdpi.com/2079-4991/11/2/483/s1, Table S1: Compound name, CAS number and chemical formula of the metabolites detected in all samples, Table S2: Identification data for MS/MS and NMR spectroscopy of the studied metabolites, Table S3: Metabolites detected in control larvae and larvae treated with 138, 275 and 555 μg mL^−1^ of non-doped CNDs, Table S4: Metabolites detected in control larvae and larvae treated with 100, 200 and 400 μg mL^−1^ of N-doped CNDs, Table S5: Metabolites detected in control larvae and larvae treated with 38, 75 and 150 μg mL^−1^ of N,S-codoped CNDs. Figure S1: HRTEM images of (A) non-doped, (B) N-doped, and (C) N,S-codoped CNDs and the respective size distributions. Figure S2: FTIR spectrum of non-doped CNDs. Figure S3: FTIR spectrum of N-doped CNDs. Figure S4: FTIR spectrum of N,S-codoped CNDs. Figure S5: Zeta potential of non-doped CNDs. Figure S6: Zeta potential of N-doped CNDs. Figure S7: Zeta potential of N,S-codoped CNDs.
