# Supplementary material for: Metabolomic Profiling Unveils the Impact of Non-Doped and Heteroatom-Doped Carbon Nanodots on Zebrafish (Danio rerio) Embryos
Source: Nanomaterials (Basel). 2021 Feb 14;11(2):483. doi: 10.3390/nano11020483 (PMC7918839; doi:10.3390/nano11020483)
Supplement: Supplementary file 1 [file nanomaterials-11-00483-s001.pdf]

Supporting information

Metabolomic Profiling Unveils the Impact of Non-doped and Heteroatom-doped Carbon Nanodots on Zebrafish (*Danio rerio*) Embryos

Theodoros G. Chatzimitakos [1], Claire Pliatsika [1], Ieremias Chousidis [2], Ioannis D. Leonardos [2] and Constantine D. Stalikas [1,*]

[1] Laboratory of Analytical Chemistry, Department of Chemistry, University of Ioannina, 45110, Ioannina, Greece; chatzimitakos@outlook.com (T.G.C.), claire.dchemrid@gmail.com (C.P.)
[2] Laboratory of Zoology, Department of Biological Applications and Technologies, University of Ioannina, 45110, Ioannina, Greece; jchousidis@gmail.com (I.C.), ileonard@uoi.gr (I.L.)
* Correspondence: cstalika@uoi.gr; Tel.: +0030-26510-08414

1. Instruments

^{1}H-NMR spectra were recorded on a Bruker AV-500 spectrometer equipped with a TXI cryoprobe (Bruker BioSpin, Rheinstetten, Germany) and spectra were processed with the TopSpin 2.1 software (Copyright 2009, Bruker BioSpin). To obtain the ^{1}H-NMR spectra, the following parameters were used: Acquisition time: 3.171 s, relaxation delay 5 s, data points: 64 K, pulse length: 90° and scans: 256. Fourier transform was applied to the spectra prior phase and baseline manual correction. Prior to MS confirmatory analyses, metabolites were separated using an Ultimate 3000 HPLC (Dionex, Milan, Italy) ultra-high-performance liquid chromatographic (UHPLC) system. A Hypersil GOLD 1.9 μm particle size (100 mm × 2.1 mm I.D.) chromatographic column was used. The oven temperature was kept constant at 30 °C. The mobile phase used consisted of water (A) and acetonitrile (B), both acidified with formic acid 0.1% (v/v). Metabolites were separated using the following gradient program: 0–13.78 min, 20–90% B, 13.78–15.28 min, 90% B, 15.28–18.06 min, 90–20% B, followed by a 2-min re-equilibration time of the column. The flow rate of the mobile phase was 300 μL min^{-1}. After the chromatographic separation, metabolites were detected using a linear trap quadrupole (LTQ) Orbitrap mass spectrometer (Thermo Scientific, Bremen, Germany), equipped with an atmospheric pressure interface and an ESI ion source. Effluents from the chromatographic system were delivered to the ion source with nitrogen as the sheath and auxiliary gas. Ionization was carried out both in positive and negative modes. For the positive ionization mode, the sample injection volume was 2.5 μL, the source voltage was 3.40 kV, the tube lens was 110 V, while the heated capillary voltage was 40.00 V and temperature was maintained at 320 °C. For the negative ionization mode, 10.0 μL samples were injected in the system, source voltage was 3.70 kV, tube lens was 120 V, the heated capillary voltage was -30.00 V, and temperature was maintained at 320 °C. Two scan modes were used in both cases. First, a full scan mode, at a resolution of 60,000 and an m/z range of 50–1500. Then, the most-intense-ion scan (MS/MS fragmentation of the most abundant ion) with a resolution of 7500 was used. The system was controlled by the Thermo Xcalibur 2.1 software (Copyright 1998-2009, Thermo Fischer Scientific Inc.). The FTIR spectra were recorded using a Perkin Elmer Spectrum Two FTIR using an attenuated total reflectance accessory (PerkinElmer, MA, USA). High-resolution transmission electron microscopy (HR-TEM) images were obtained with a JEOL JEM-2100 microscope operated at 200 kV equipped with LaB6 filament. Zeta-potential measurements (average of 3 measuring cycles of 100 repetitions each) were performed on suspension of the carbon dots in commercially available purified water using a Nano Zetasizer (Nano ZS, Malvern, UK). A disposable DTS 1070 folded capillary cell was used for the measurements. Solutions of the nanomaterials were adjusted to pH 7.0 and

Citation: Chatzimitakos, T.G.; Pliatsika, C.; Chousidis, I.; Leonardos, I.D. Stalikas, C.D. Metabolomic Profiling Unveils the Impact of Non-doped and Heteroatom-doped Carbon Nanodots on Zebrafish (Danio rerio) Embryos. *Nanomaterials* **2021**, *11*, x. https://doi.org/10.3390/xxxxx

Academic Editor: Robyn L. Tanguay

Received: 12 January 2021
Accepted: 11 February 2021
Published: 14 February 2021

Publisher's Note: MDPI stays neutral with regard to jurisdictional claims in published maps and institutional affiliations.

Copyright: © 2021 by the authors. Submitted for possible open access publication under the terms and conditions of the Creative Commons Attribution (CC BY) license (http://creativecommons.org/licenses/by/4.0/).

measured at 25 °C, while no other physicochemical parameters of the solutions were adjusted.

2. CND characterization

The synthesized CNDs were characterized by recording their FTIR spectra, obtaining HRTEM images, measuring their zeta potential, and comparing the results with previous studies [4,5]. As can be seen in Figure S1, all three CND species have a circular shape, and their size distribution is between 4 nm and 6 nm. As regards the FTIR spectra (Figure S2, S3, and S4), in all cases a wide absorption band can be observed at ~3200 cm^{-1}, which is attributed to the -O-H groups of the CNDs. In the case of non-doped CNDs, peaks were observed at 1580 (stretching vibrations of the –C=C– and -C=O groups of aromatic rings), 1390 (stretching of -C-H), 1310 (stretching of -C-H), 1195 (stretching of -C-OH), 1155, and 1075 cm^{-1} (asymmetric stretching vibrations of -C-O-C groups) [6–8]. In the spectrum of the N-doped CNDs, peaks at 1560 (bending vibration of –N–H group and/or asymmetric bending vibration of the –N–O group), 1415 (bending vibration of the –C–H in case of alkanes), 1320 (bending vibration of the –C–O group), and 1175 cm^{-1} (-C-N stretching) were recorded. Moreover, a small peak appeared close to 990 cm^{-1}, which can be attributed to the stretching vibration of the –C–N in the case of aliphatic amines [9–11]. Finally, with respect to the spectrum of the N,S-codoped CNDs, peaks at 3160, 2060 (stretching vibration of S-H), 1680 (stretching vibration of –C=N), 1590 (bending vibration of the –N–O), 1385 (stretching vibration of the –C–N in case of aromatic amines), 1290 (-C-N and N-H stretching), 1180 (vibration of the –C–H group), and 1085 cm^{-1} (stretching vibration of the –C–N in case of aliphatic amines) were recorded [12]. In the cases of N-doped and N,S-doped CNDs, successful doping of the CNDs with the heteroatoms was confirmed by the appearance of characteristic peaks on the spectra. Moreover, the spectra were similar to those of our previous report [5]. Finally, the zeta potential of the CNDs was recorded in water (pH 7), and the results are depicted in Figures S5, S6, and S7. The zeta potential of non-doped CNDs, N-doped, and N,S-codoped CNDs was -6 mV, -37 mV, and -20 mV, respectively. Zeta potential was also recorded in E3 medium, and an insignificant change was recorded (-8 mV, -34 mV, and -24 mV for non-doped, N-doped, and N,S-doped CNDs, respectively). The hydrodynamic diameter in the E3 medium was ~7.5 nm for all three CNDs. This was anticipated, since the hydrodynamic diameter of a nanomaterial is generally larger than its real size [13]. Thus, no change of CNDs was attributed to interactions with E3 medium.

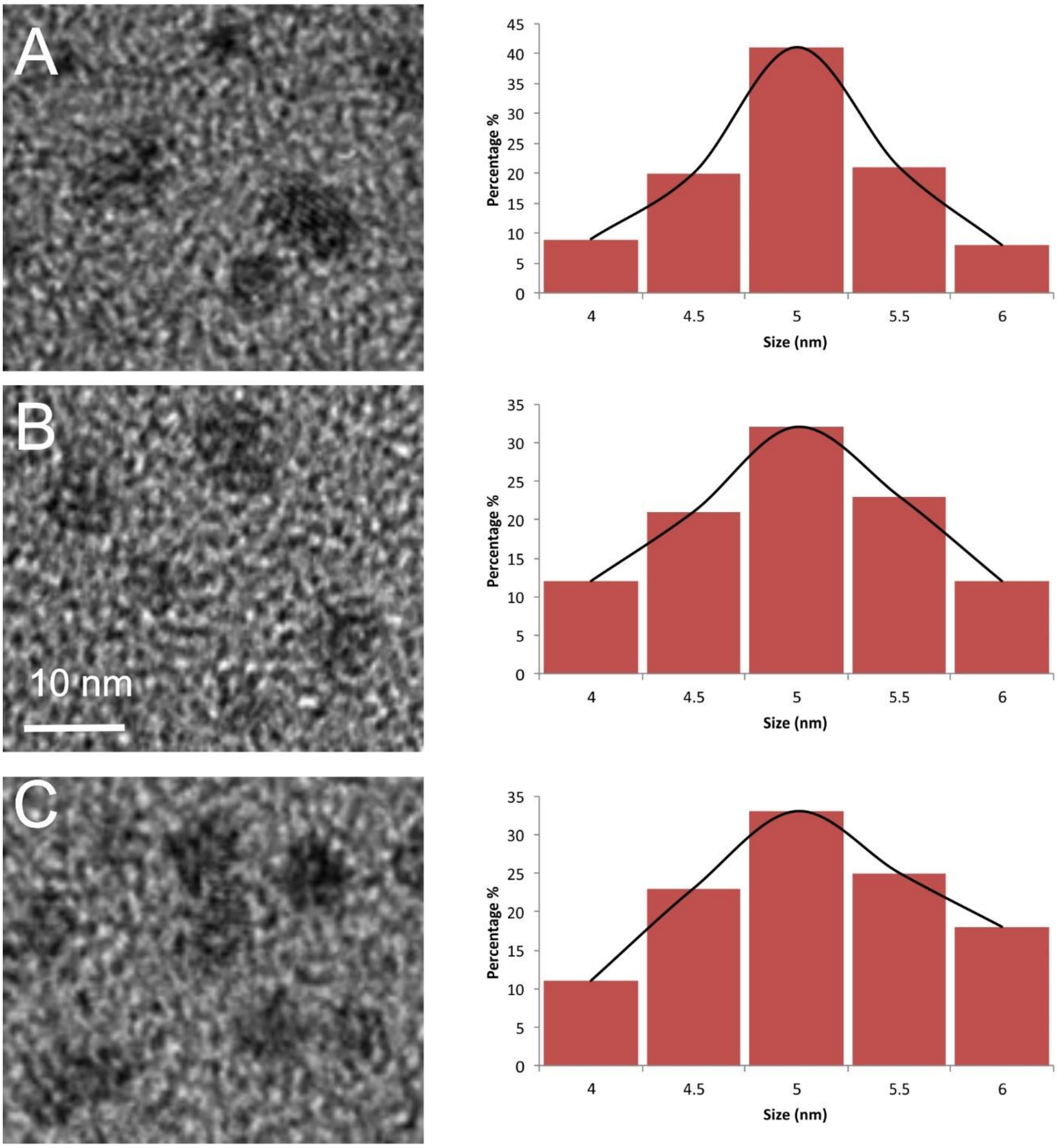

Figure S1. HRTEM images of (**A**) non-doped, (**B**) N-doped, and (**C**) N,S-doped CNDs and their respective size distributions.

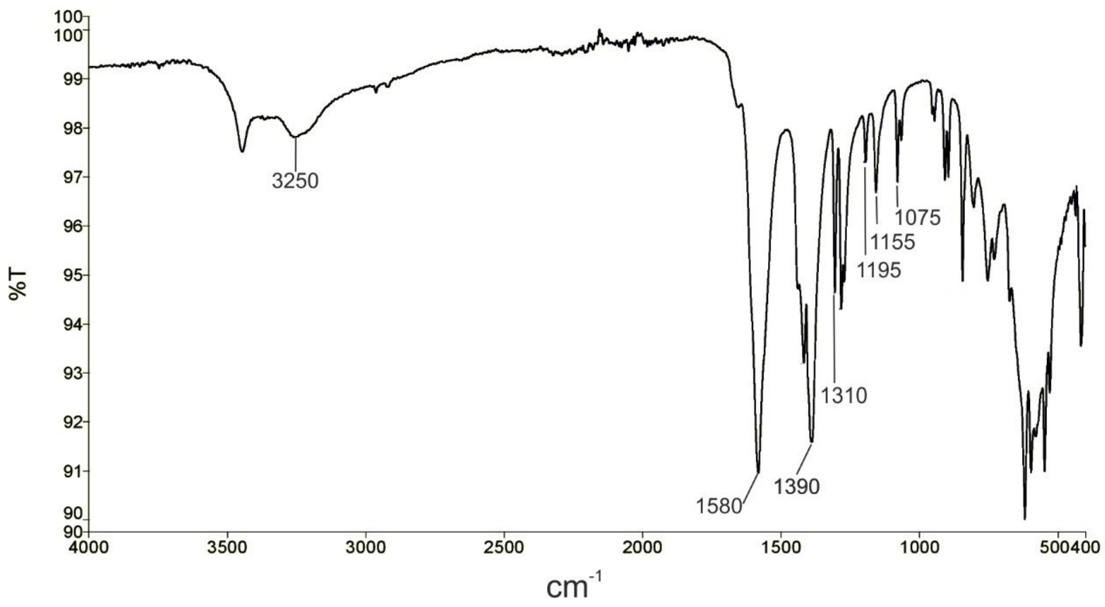

Figure S2. FTIR spectrum of non-doped CNDs.

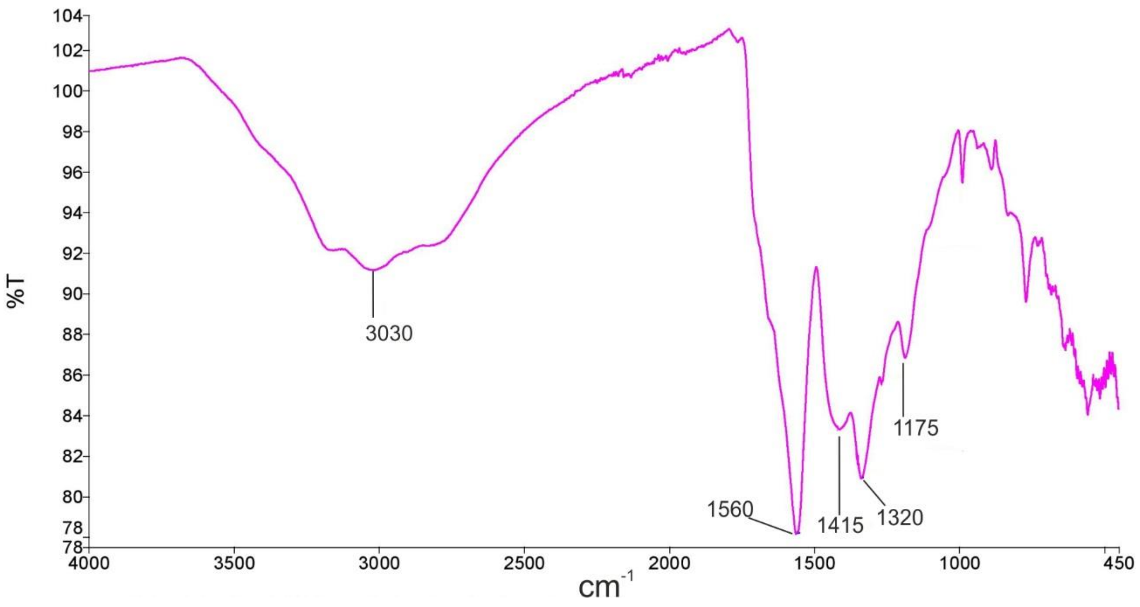

Figure S3. FTIR spectrum of N-doped CNDs.

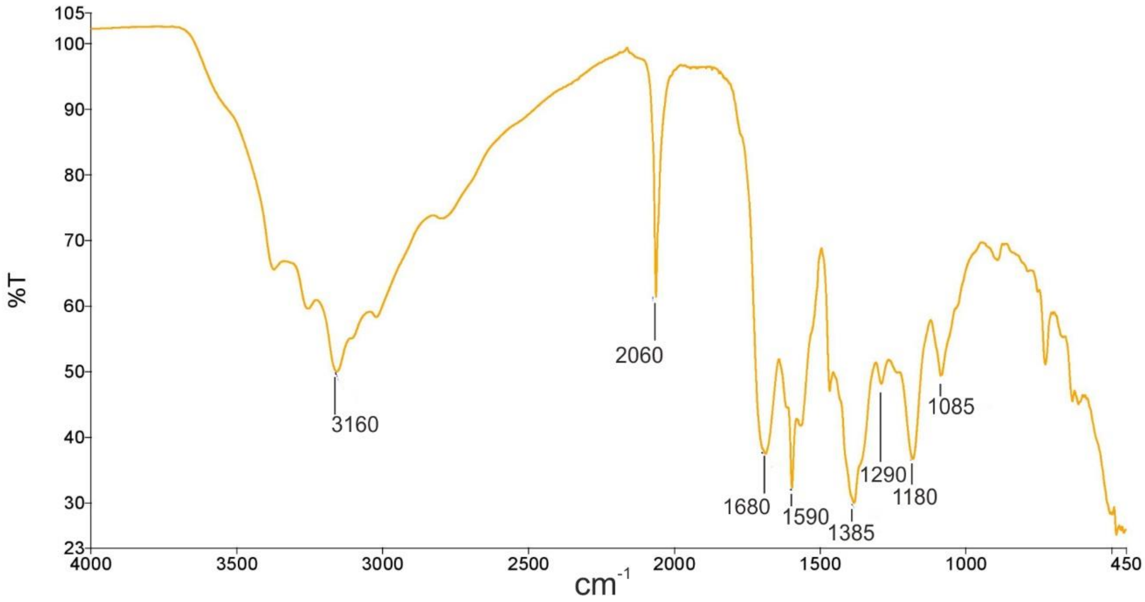

Figure S4. FTIR spectrum of N,S-codoped CNDs.

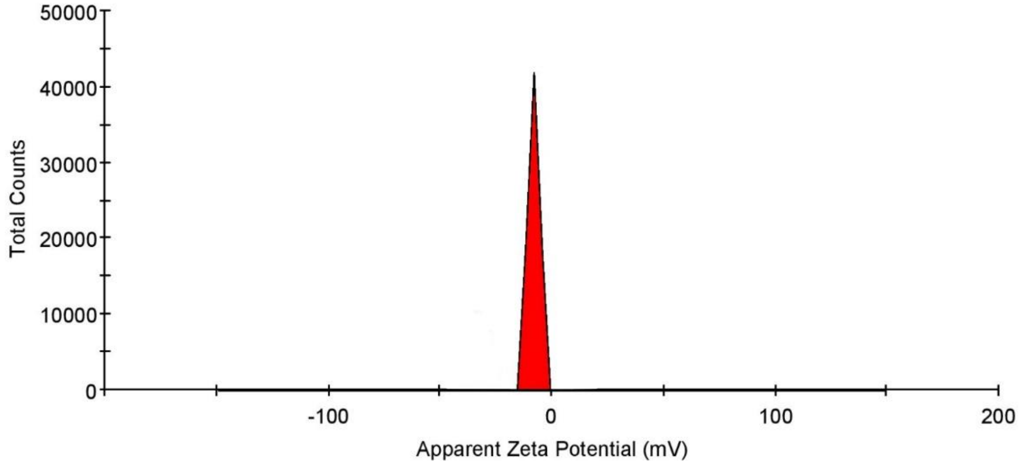

Figure S5. Zeta potential of non-doped CNDs.

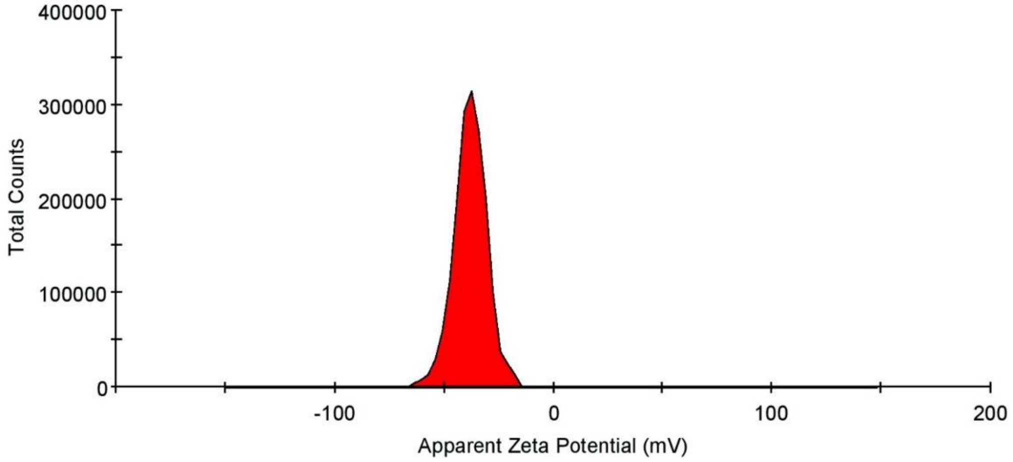

Figure S6. Zeta potential of N-doped CNDs.

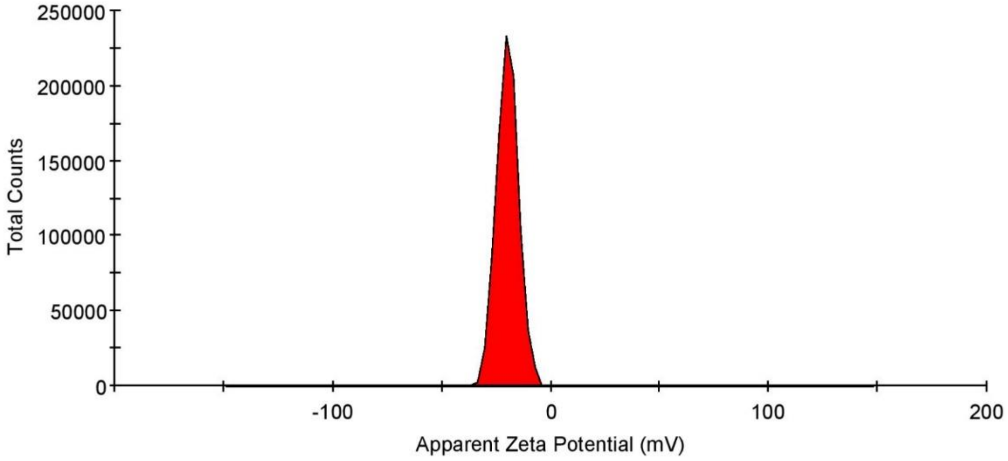

Figure S7. Zeta potential of N,S-codoped CNDs.

Table S1. Compound name, CAS number, and chemical formula of the metabolites detected in all samples.

Metabolite	CAS number	Chemical formula
α-D-glucose	492-62-6	$C_6H_{12}O_6$
α-Ketoisovaleric acid	759-05-7	$C_5H_8O_3$
α-Lactose	63-42-3	$C_{12}H_{22}O_{11}$
Biotin	58-85-5	$C_{10}H_{16}N_2O_3S$
Citrulline	372-75-8	$C_6H_{13}N_3O_3$
D-fructose	53188-23-1	$C_6H_{12}O_6$
D-Maltose	69-79-4	$C_{12}H_{22}O_{11}$
D-Mannose	3458-28-4	$C_6H_{12}O_6$
D-Xylose	58-86-6	$C_5H_{10}O_5$
Fructose 6-phosphate	643-13-0	$C_6H_{13}O_9P$
γ-aminobutyric acid	56-12-2	$C_4H_9NO_2$
Glucosamine 6-phosphate	3616-42-0	$C_6H_{14}NO_8P$
Glutathione	70-18-8	$C_{10}H_{17}N_3O_6S$
Glycerol 3-phosphate	57-03-4	$C_3H_9O_6P$
Inosine	58-63-9	$C_{10}H_{12}N_4O_5$
L-Arabitol	7643-75-6	$C_5H_{12}O_5$
L-Cystathione	56-88-2	$C_7H_{14}N_2O_4S$
L-Cystine	56-89-3	$C_6H_{12}N_2O_4S_2$
L-Fucose	2438-80-4	$C_6H_{12}O_5$
L-Histidine	71-00-1	$C_6H_9N_3O_2$
L-Isoleucine	73-32-5	$C_6H_{13}NO_2$
L-Methionine	63-68-3	$C_5H_{11}NO_2S$
L-Proline	147-85-3	$C_5H_9NO_2$
L-Tyrosine	60-18-4	$C_9H_{11}NO_3$
Melibiose	585-99-9	$C_{12}H_{22}O_{11}$
Pipecolic acid	535-75-1	$C_6H_{11}NO_2$
Rafinose	512-69-6	$C_{18}H_{32}O_{16}$
S- Adenosylmocysteine	979-92-0	$C_{14}H_{20}N_6O_5S$
Selenomethionine	3211-76-5	$C_5H_{11}NO_2Se$
Stachyose	470-55-3	$C_{24}H_{42}O_{21}$
Sucrose	57-50-1	$C_{12}H_{22}O_{11}$
Trehalose	99-20-7	$C_{12}H_{22}O_{11}$
Glycuronic acid uridine diphosphate	2616-64-0	$C_{15}H_{22}N_2O_{18}P_2$
Uridine N-acetylglucosamine diphosphate	528-04-1	$C_{17}H_{27}N_3O_{17}P_2$

Table S2. Identification data for MS/MS and NMR spectroscopy of the studied metabolites.

Metabolite	Theoretical mass	Experimental mass	Difference (ppm)	MS/MS fragments	^{1}H peaks
α-D-glucose	181.1632	181.163	-1,1	163.0600/145.0495/103.0389 /91.0389	3.52 (dd, *J*=9.82, 3.77 Hz)
α-Ketoisovaleric acid	117.1225	117.1226	0,85	99.044/71.0491	1.11(d, J=7.14 Hz)
α-Lactose	343.3038	343.3036	-0,58	181.0706/163.0600/147.0651 /119.0338	3.59 (d, J=3.68 Hz)
Biotin	245.3183	245.3184	0,41	210.058/199.0899/157.0430	2.20 (t, J=7.43 Hz)
Citrulline	176.193	176.1929	-0,57	116.0706/85.0760/70.0651	3.74 (dd, *J*=6.42, 5.84 Hz)
D-fructose	180.1559	180.1559	2.22	243.0264/171.0053/147.0651	4.01 (dd, J = 4.5, 3.4 Hz), 4.46 (d, J = 4.5 Hz)
D-Maltose	343.3038	343.3037	-0,29	281.0867/181.0706/163.0600 /145.0495	3.64-3.68 (m), 5.40 (d, J=3.89 Hz)
D-Mannose	181.1632	181.1634	1,1	145.0495/103.0389/91.0389	5.17 (d, J=1.31 Hz)
D-Xylose	151.1372	151.1374	1,32	115.0389/91.0389	4.41 (d, J=10.26 Hz)
Fructose 6-phosphate	261.1431	261.1431	0	147.0651/110.9841/96.9685	3.60-3.70 (m)
γ-aminobutyric acid	104.1271	104.1272	0,96	87.044/77.0597	2.28 (t, J=7.36 Hz)
Glucosamine 6-phosphate	260.1583	260.1584	0,38	171.0053/146.0811/103.0389	5.45 (d, J=3.57 Hz)
Glutathione	308.3303	308.3304	0,32	228.0978/187.0535/159.0586 /102.0549	4.20 (q, J=7.14, 7.14, 7.14 Hz)
Glycerol 3-phosphate	173.081	173.0809	-0,58	136.9998/96.9685/75.0440	3.61 (dd, J=11.80, 5.90 Hz)
Inosine	269.2334	269.2333	-0,37	199.0713/137.0457/120.0192	8.18 (s), 8.30 (s)
L-Arabitol	153.1531	153.1532	0,65	135.0651/117.0546/75.0440	3.63-3.69 (m)
L-Cystathione	223.2693	223.2693	0	177.0692/160.0426/148.0426	3.85 (dt, J=6.85, 5.56 Hz)
L-Cystine	241.3073	241.3074	0,41	177.9990/149.0201/119.9936	3.18 (dd, J=14.94, 8.12 Hz)
L-Fucose	165.1638	165.1638	0	129.0546/105.0546/87.0440	5.21 (d, J=3.90 Hz)
L-Histidine	156.1619	156.162	0,64	139.0502/110.0712/93.0	7.09 (d, J=0.58 Hz)

				447	
L-Isoleucine	132.1802	132.1804	1,51	114.0913/86.0964	1.240-1.254 (m)
L-Methionine	150.2183	150.2184	0,67	104.0528/88.0215	2.05-2.24 (m)
L-Proline	116.1378	116.1379	0,86	98.0600/72.0807	1.99-2.06 (m), 4.12 (dd, J=8.63, 6.42 Hz)
L-Tyrosine	182.1958	182.1959	0,55	164.0706/136.0756/109.0647	6.86-6.90 (m)
Melibiose	343.3038	343.3038	0	307.1023/281.0867/253.0917	3.68-3.78 (m)
Pipecolic acid	130.1643	130.1644	0,77	112.0756/94.0651/84.0807	1.49-1.75 (m), 2.17-2.30 (m)
Rafinose	505.4444	505.4447	0,59	325.1129/181.0706/163.0600 /93.0546	4.12 (d, J=6.30 Hz)
S-Adenosylmocysteine	385.4183	385.4181	-0,52	220.0638/136.0617/119.0352	1.78-1.84 (m), 8.37 (s)
Selenomethionine	197.1173	197.1176	1,52	151.9972/122.9707/120.9550	1.72 (q, J=7.5, 7.3, 7.5 Hz)
Stachyose	667.585	667.5848	-0,3	649.2185/487.1657/325.1129 /181.0706/163.0600	4.60 (d, J=2.64 Hz)
Sucrose	343.3038	343.3038	0	181.0706/163.0600/121.0495 /105.0546	4.21 (d, J=8.75 Hz), 5.40 (d, J=3.89 Hz)
Trehalose	343.3038	343.3036	-0,58	325.1129/281.0867/181.070 /163.0600/119.0338	3.64 (dd, *J*=9.93, 3.84 Hz), 3.85 (s)
Glycuronic acid uridine diphosphate	580.2853	580.2859	1.03	377.0145/227.0.662/113.0345	6.21 (d, J = 9.3 Hz), 7.61 (d, J = 10.8 Hz)
Udirine N-acetylglucosamine diphosphate	608.361	608.3608	-0,33	539.0673/364.0193/284.0529 /113.0345/83.0239	2.07 (s), 5.50 (dd, *J*=7.14, 3.29 Hz)

Table S3. Metabolites detected in control larvae and larvae treated with 138, 275, and 555 μg mL^{-1} of non-doped CNDs; + denotes active metabolic pathways in the sample, while – denotes down-regulated pathways.

Metabolite	Control	138 μg mL^{-1} ($LC_{50/4}$)	275 μg mL^{-1} ($LC_{50/2}$)	550 μg mL^{-1} (LC_{50})
Alpha-D-Glucose	+	-	-	-
D-Fructose	+	-	-	-
D-Mannose	+	-	-	-
Fructose 6-phosphate	+	-	-	-
Glucosamine 6-phosphate	+	-	-	-
Raffinose	+	-	-	-
Uridine diphosphate glucuronic acid	+	-	-	-
Uridine diphosphate-N-acetylglucosamine	+	-	-	-
Alpha-Lactose	+	+	-	-
Biotin	+	+	-	-
Stachyose	+	+	-	-
D-Maltose	+	+	-	+
L-Cystathionine	+	+	-	+
S-Adenosylhomocysteine	-	+	-	+
Sucrose	+	-	+	-
Citrulline	-	+	-	-
gamma-Aminobutyric acid	-	+	-	-
Glutathione	-	+	+	-
L-Fucose	-	+	-	-
L-Histidine	-	+	-	-
L-Tyrosine	-	+	-	-
Melibiose	-	+	-	-
Selenomethionine	-	+	-	-
alpha-Ketoisovaleric acid	-	-	+	-
Inosine	-	-	+	-
L-Arabitol	-	-	+	-
L-Cystine	-	-	+	-
Pipecolic acid	-	-	+	-
Trehalose	-	-	+	-

Table S4. Metabolites detected in control larvae and larvae treated with 100, 200, and 400 μg mL^{-1} of N-doped CNDs; + denotes active metabolic pathways in the sample, while – denotes down-regulated pathways.

Metabolite	Control	100 μg mL^{-1} ($LC_{50/4}$)	200 μg mL^{-1} ($LC_{50/2}$)	400 μg mL^{-1} (LC_{50})
Raffinose	+	+	+	+
Alpha-Lactose	+	+	+	+
Alpha-D-Glucose	+	+	+	+
Stachyose	+	+	+	+
D-Maltose	+	+	+	+
Fructose 6-phosphate	+	-	-	-
Glucosamine 6-phosphate	+	-	-	-
D-Mannose	+	-	-	-
Uridine diphosphate glucuronic acid	+	-	-	-
Uridine diphosphate-N-acetylglucosamine	+	-	-	-
D-Fructose	+	-	-	-
Biotin	+	-	-	-
L-Cystathionine	+	-	-	-
Sucrose	+	-	-	-
L-Isoleucine	-	-	+	-
D-Xylose	-	-	+	-
Citrulline	-	-	+	-
S-Adenosylhomocysteine	-	+	+	+

Table S5. Metabolites detected in control larvae and larvae treated with 38, 75, and 150 μg mL^{-1} of N,S-codoped CNDs; + denotes active metabolic pathways in the sample, while – denotes pathways that are down-regulated pathways.

Metabolite	Control	38 μg mL^{-1} ($LC_{50/4}$)	75 μg mL^{-1} ($LC_{50/2}$)	150 μg mL^{-1} (LC_{50})
D-Maltose	+	+	+	+
Glucosamine 6-phosphate	+	+	+	+
Raffinose	+	+	+	-
Alpha-D-Glucose	+	+	+	-
Stachyose	+	+	+	-
Fructose 6-phosphate	+	+	+	-
D-Mannose	+	+	+	-
L-Cystathionine	+	+	+	-
Sucrose	+	+	+	-
Alpha-Lactose	+	+	-	+
Uridine diphosphate glucuronic acid	+	-	-	-
Uridine diphosphate-N-acetylglucosamine	+	-	-	-
D-Fructose	+	-	-	-
Biotin	+	-	-	-
L-Isoleucine	-	+	-	-
L-Proline	-	+	-	-
D-Xylose	-	+	+	-
Melibiose	-	+	+	-
S-Adenosylhomocysteine	-	-	-	+
L-Arabitol	-	-	+	-

References

1. Westerfield, M. The Zebrafish Book. A Guide for the Laboratory Use of Zebrafish (Danio rerio), 5th Edition. *Univ. Oregon Press. Eugene* **2007**.
2. Wilhelm, K.-P.; Zhai, H.; Maibach, H.I.; Wilhelm, K.-P.; Maibach, H.I. OECD guidelines for testing of chemicals. In *Dermatotoxicology*; 2012; pp. 497–499.
3. Kimmel, C.B.; Ballard, W.W.; Kimmel, S.R.; Ullmann, B.; Schilling, T.F. Stages of embryonic development of the zebrafish. *Dev. Dyn.* **1995**, *203*, 253–310, doi:10.1002/aja.1002030302.
4. Chousidis, I.; Stalikas, C.D.; Leonardos, I.D. Induced toxicity in early-life stage zebrafish (Danio rerio) and its behavioral analysis after exposure to non-doped, nitrogen-doped and nitrogen, sulfur-co doped carbon quantum dots. *Environ. Toxicol. Pharmacol.* **2020**, *79*, doi:10.1016/j.etap.2020.103426.
5. Chatzimitakos, T.G.; Kasouni, A.I.; Troganis, A.N.; Stalikas, C.D. Exploring the antibacterial potential and unraveling the mechanism of action of non-doped and heteroatom-doped carbon nanodots. *J. Nanoparticle Res.* **2020**, *22*, doi:10.1007/s11051-019-4736-6.

6. Wang, Y.; Wu, W.T.; Wu, M.B.; Sun, H. Di; Xie, H.; Hu, C.; Wu, X.Y.; Qiu, J.S. Yellow-visual fluorescent carbon quantum dots from petroleum coke for the efficient detection of Cu2+ ions. *Xinxing Tan Cailiao/New Carbon Mater.* **2015**, doi:10.1016/S1872-5805(15)60204-9.
7. Yang, K.; Liu, M.; Wang, Y.; Wang, S.; Miao, H.; Yang, L.; Yang, X. Carbon dots derived from fungus for sensing hyaluronic acid and hyaluronidase. *Sensors Actuators, B Chem.* **2017**, *251*, 503–508, doi:10.1016/j.snb.2017.05.086.
8. Macairan, J.R.; Zhang, I.; Clermont-Paquette, A.; Naccache, R.; Maysinger, D. Ratiometric pH Sensing in Living Cells Using Carbon Dots. *Part. Part. Syst. Charact.* **2020**, *37*, doi:10.1002/ppsc.201900430.
9. Chatzimitakos, T.; Kasouni, A.; Sygellou, L.; Avgeropoulos, A.; Troganis, A.; Stalikas, C. Two of a kind but different: Luminescent carbon quantum dots from Citrus peels for iron and tartrazine sensing and cell imaging. *Talanta* **2017**, *175*, 305–312, doi:10.1016/j.talanta.2017.07.053.
10. Chatzimarkou, A.; Chatzimitakos, T.G.; Kasouni, A.; Sygellou, L.; Avgeropoulos, A.; Stalikas, C.D. Selective FRET-based sensing of 4-nitrophenol and cell imaging capitalizing on the fluorescent properties of carbon nanodots from apple seeds. *Sensors Actuators, B Chem.* **2018**, *258*, 1152–1160, doi:10.1016/j.snb.2017.11.182.
11. Chatzimitakos, T.G.; Kasouni, A.I.; Troganis, A.N.; Stalikas, C.D. Carbonization of Human Fingernails: Toward the Sustainable Production of Multifunctional Nitrogen and Sulfur Codoped Carbon Nanodots with Highly Luminescent Probing and Cell Proliferative/Migration Properties. *ACS Appl. Mater. Interfaces* **2018**, *10*, 16024–16032, doi:10.1021/acsami.8b03263.
12. Chatzimitakos, T.; Kasouni, A.; Sygellou, L.; Leonardos, I.; Troganis, A.; Stalikas, C. Human fingernails as an intriguing precursor for the synthesis of nitrogen and sulfur-doped carbon dots with strong fluorescent properties: Analytical and bioimaging applications. *Sensors Actuators, B Chem.* **2018**, *267*, 494–501, doi:10.1016/j.snb.2018.04.059.
13. Dager, A.; Uchida, T.; Maekawa, T.; Tachibana, M. Synthesis and characterization of Mono-disperse Carbon Quantum Dots from Fennel Seeds: Photoluminescence analysis using Machine Learning. *Sci. Rep.* **2019**, *9*, doi:10.1038/s41598-019-50397-5.
